# Supplementary material for: A termination-insensitive and robust electron gas at the heterointerface of two complex oxides
Source: Nat Commun. 2019 Sep 6;10:4026. doi: 10.1038/s41467-019-12036-5 (PMC6731279; doi:10.1038/s41467-019-12036-5)
Supplement: Supplementary file 1 — Supplementary Information [file 41467_2019_12036_MOESM1_ESM.pdf]

Supplementary information for

A termination-insensitive and robust electron gas at the heterointerface of two complex oxides

Zhang et al.

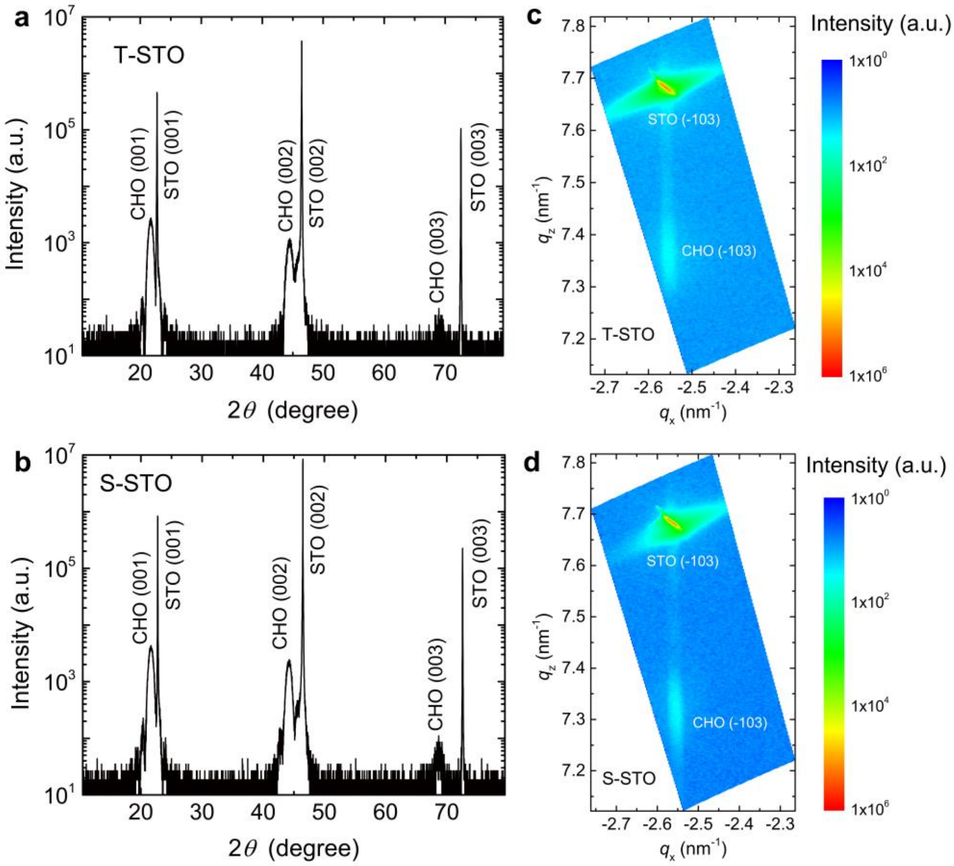

Supplementary Figure 1 | Structural characterization by x-ray diffraction (XRD). a, b, Out-of-plane θ-2θ XRD patterns for 20 uc $CaHfO_3$ (CHO) grown on TiO_2-terminated $SrTiO_3$ (T-STO) and SrO-terminated $SrTiO_3$ (S-STO), respectively. **c, d,** Reciprocal space maps (RSM) about the STO (-103) condition, for 20 uc CHO grown on T-STO and S-STO, respectively.

The x-ray diffraction (XRD) reciprocal space maps (RSM) (Supplementary Figure 1c-d) indicate that for both TiO_2-terminated $SrTiO_3$ (T-STO) and SrO-terminated $SrTiO_3$ (S-STO), the $CaHfO_3$ (CHO) films are constrained on the STO substrates, with an in-plane lattice constant of 3.905 Å and an out-of-plane lattice constant of 4.075 Å for CHO/T-STO (or 4.102 Å for CHO/S-STO) (the bulk pseudocubic or cubic lattice constant of CHO and STO are 3.990 and 3.905 Å, respectively). Therefore, in both cases, the CHO films are under a compressive strain, which is contrary to the case of $LaAlO_3$/STO, in which the $LaAlO_3$ film is under a tensile strain (the bulk pseudocubic lattice constant of $LaAlO_3$ is 3.789 Å).

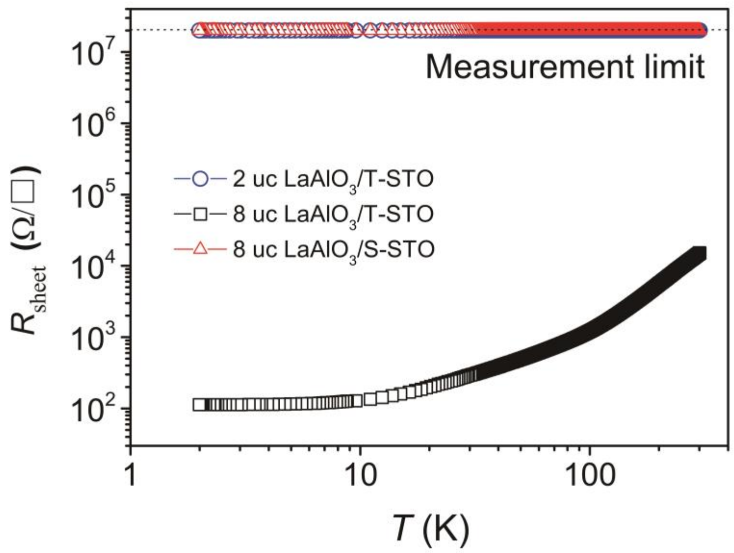

Supplementary Figure 2 | Electrical transport properties of $LaAlO_3/SrTiO_3$ heterointerfaces. These samples were prepared under the same growth conditions as the $CaHfO_3/SrTiO_3$ heterointerfaces presented in Figs. 2a and 2b in the main text, without post annealing in O_2.

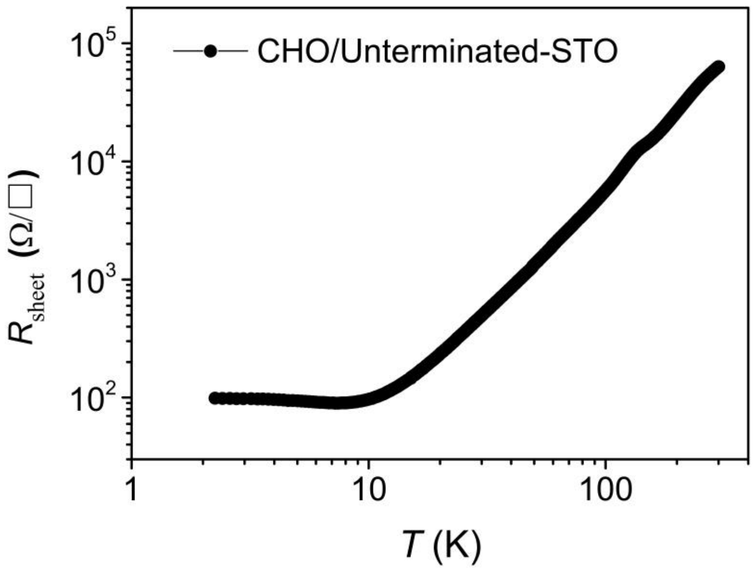

Supplementary Figure 3 | Electrical transport property of an 8uc $CaHfO_3/SrTiO_3$ heterointerface grown on unterminated $SrTiO_3$.

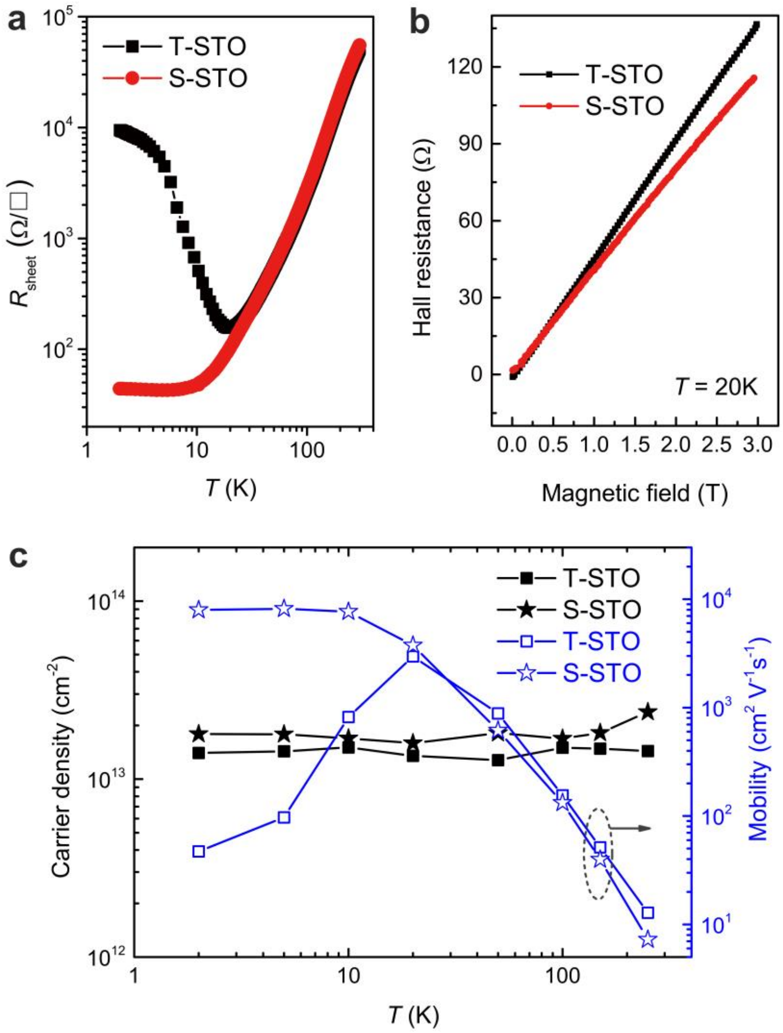

Supplementary Figure 4 | Hall effect measurements of two typical $CaHfO_3/SrTiO_3$ (CHO/STO) heterointerfaces. The two samples are 8uc CHO grown on TiO_2-termianted STO (T-STO) and SrO-terminated STO (S-STO), respectively. They were grown using the conditions as described in the **Methods** of the main text, and followed by an in situ annealing under $P(O_2)$ = 200 mbar, at a temperature of 500 ℃ for 30 minutes. **a,** temperature dependence of R_{sheet}. The low-temperature upturn of the R_{sheet}-T curve for the 8uc CHO/T-STO might be attributed to Kondo scattering. Similar observations have been reported in $LaAlO_3$/STO[1–3] and electrolyte gated STO[4]. **b,** Hall resistance versus magnetic field at 20 K, showing that both heterointerfaces have n-type conductivity. **c**, Temperature dependence of carrier density (black symbols) and mobility (blue symbols) of the two heterointerfaces.

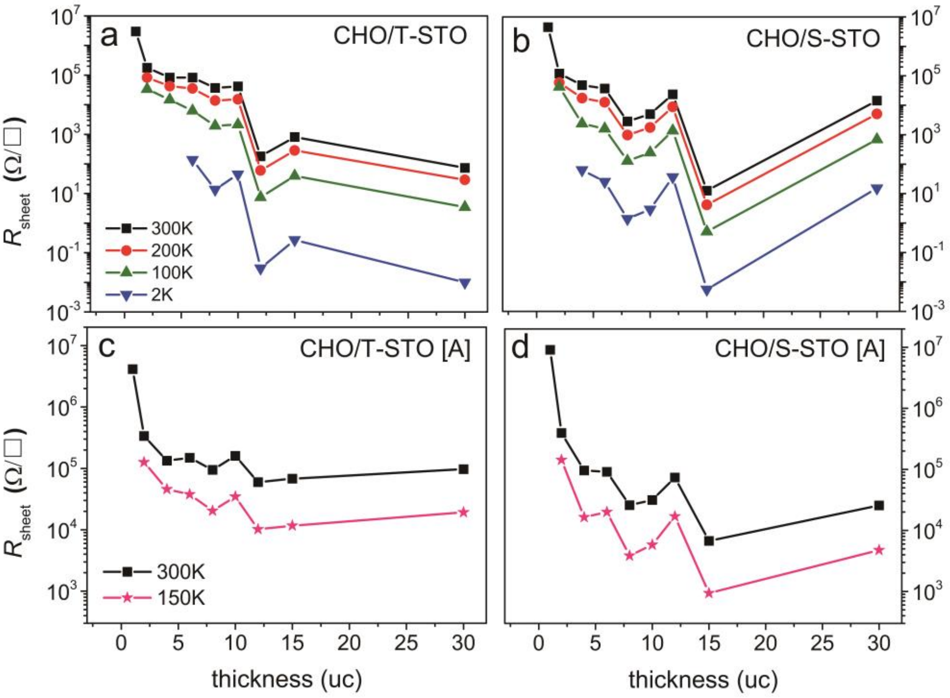

Supplementary Figure 5 | R_{sheet} as a function of $CaHfO_3$ thickness for samples of both terminations. a and **b** are regenerated from the data shown in Figs. 2a and 2b in the main text, respectively. **c** and **d** are regenerated from the data shown in Supplementary Figure 6a-b, respectively. [A] denotes that the samples were post annealed in O_2.

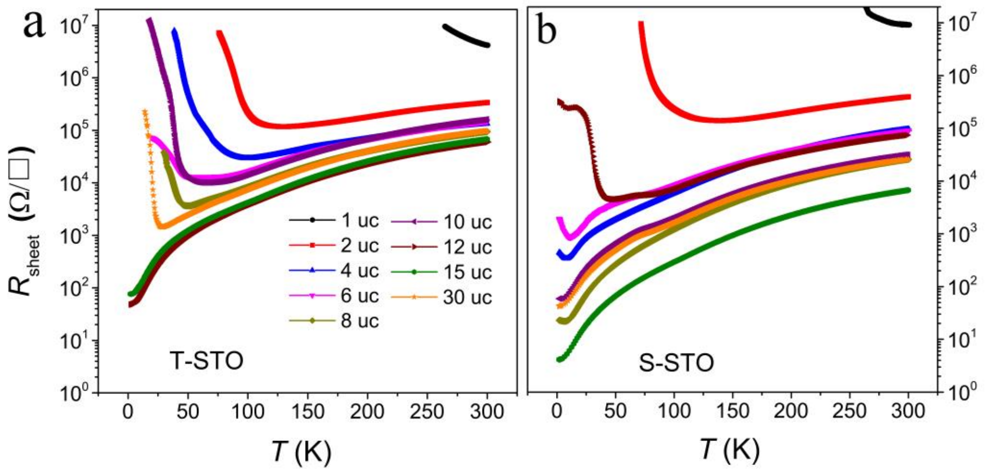

Supplementary Figure 6 | Electrical transport properties of annealed $CaHfO_3$/$SrTiO_3$ (CHO/STO) heterointerfaces. a, b, Temperature dependence of R_{sheet} for the annealed CHO of various thicknesses grown on TiO_2-terminated STO (T-STO) and SrO-terminated STO (S-STO), respectively. The samples were post annealed ex situ in a tube furnace under an oxygen partial pressure of $P(O_2)$ = 1 bar, at a temperature of 500 ℃ for 1 hour.

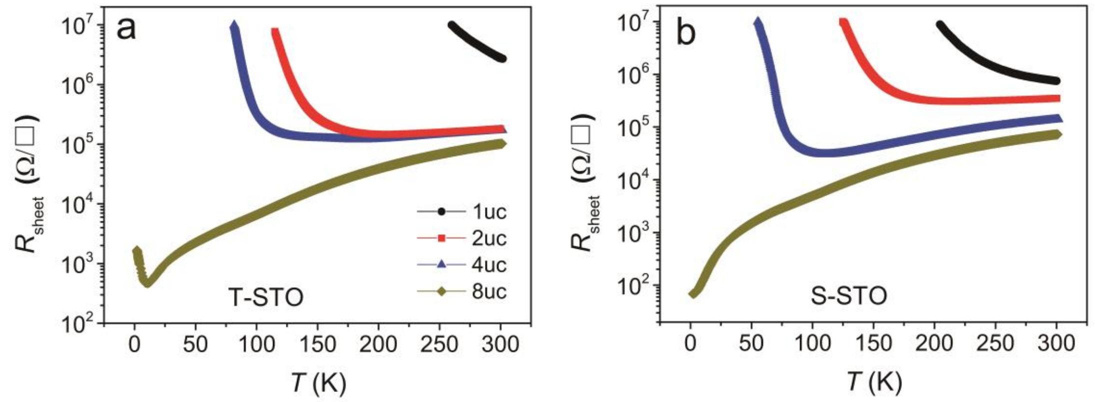

Supplementary Figure 7 | Electrical transport properties of annealed $CaHfO_3/SrTiO_3$ (CHO/STO) heterointerfaces. a, b, Temperature dependence of R_{sheet} for the annealed CHO of various thicknesses grown on TiO_2-terminated STO (T-STO) and SrO-terminated STO (S-STO), respectively. The samples were post annealed in situ under an oxygen partial pressure $P(O_2)$ = 200 mbar, at a temperature of 500 ℃ for 30 minutes.

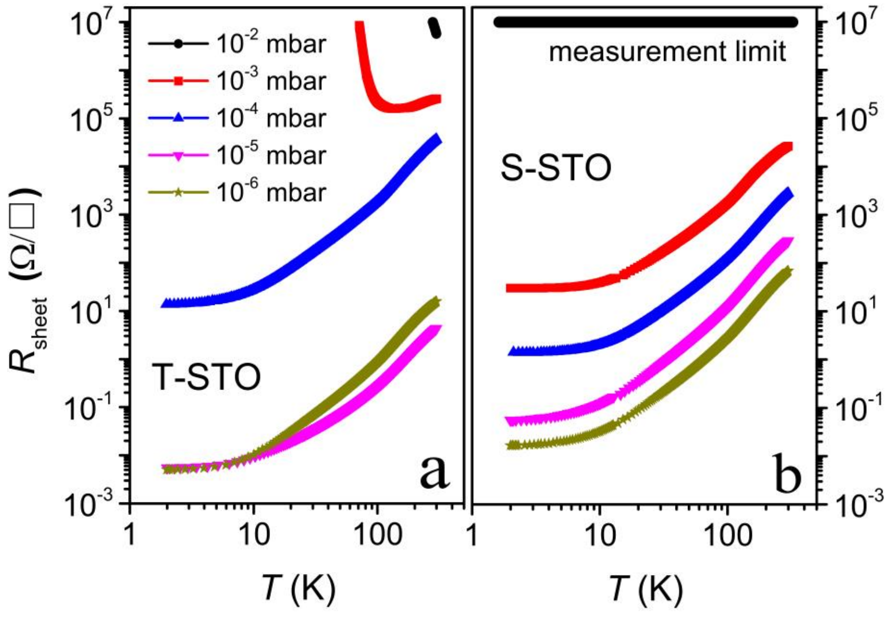

Supplementary Figure 8 | Effect of growth partial oxygen pressure. Temperature dependence of R_{sheet} for the 8 uc $CaHfO_3$ grown on (**a**) TiO_2-terminated $SrTiO_3$ (T-STO) and (**b**) SrO-terminated $SrTiO_3$ (S-STO), deposited at various partial oxygen pressures, without post annealing in O_2.

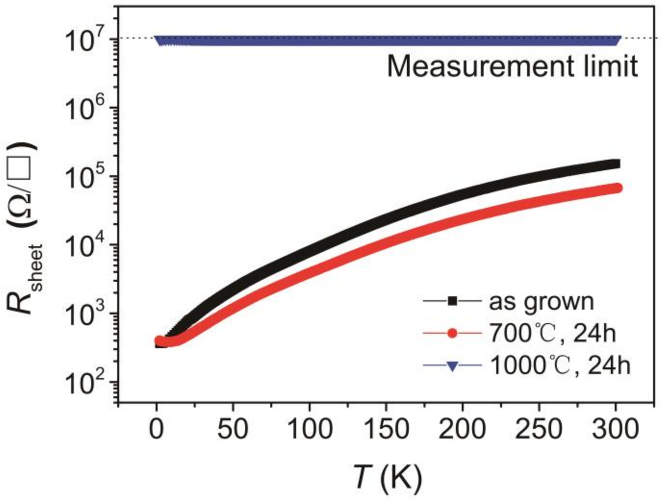

Supplementary Figure 9 | R_{sheet} as a function of temperature for a $LaAlO_3$(8 uc)/TiO_2-terminated $SrTiO_3$ heterointerface annealed under extreme condition. The as-grown sample was annealed ex situ subsequently from low to high annealing temperature, under an oxygen pressure $P(O_2)$ = 1 bar, in a tube furnace.

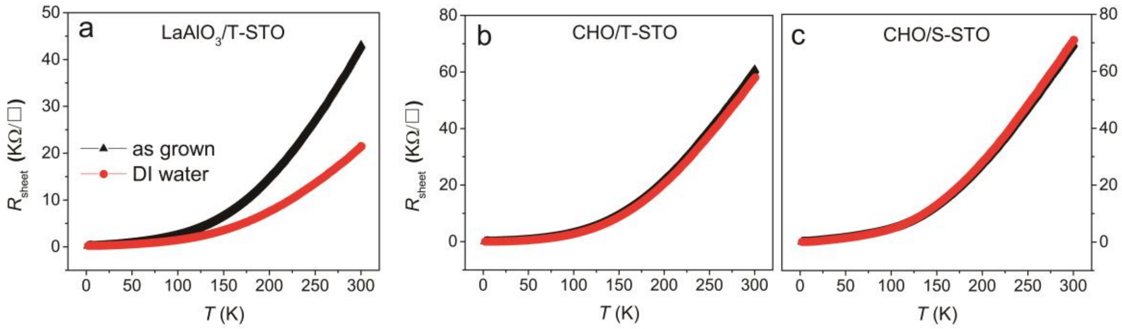

Supplementary Figure 10 | Effect of surface treatment with deionized water (DI water) on different heterointerfaces. The thickness of $LaAlO_3$ and $CaHfO_3$ is 8 uc.

polar solvents	Samples		
	$LaAlO_3$/T-STO	CHO/T-STO	CHO/S-STO
deionized water	-100.5%	-5.7%	-6.7%
ethanol	-206.5%	-7.7%	-6.6%
acetone	-209.7%	-7.5%	-5.6%

Supplementary Table 1 | Effect of surface treatment with different polar solvents. The change ratio is defined as [R_{sheet}(polar)- R_{sheet}(initial)]/R_{sheet}(polar). The thickness of $LaAlO_3$ and $CaHfO_3$ is 8 uc. The data were measured at room temperature.

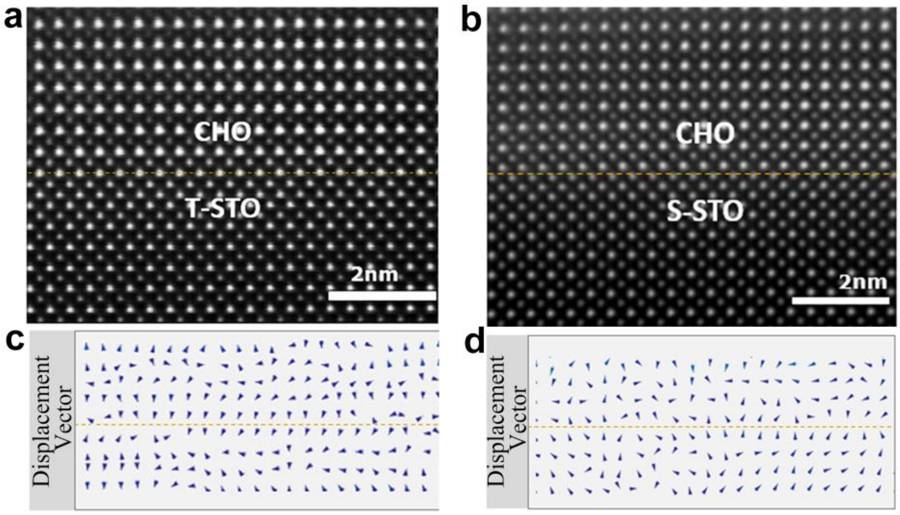

Supplementary Figure 11 | B-site atom displacement vector maps. a, b, Scanning transmission electron microscopy (STEM) images of $CaHfO_3/TiO_2$-terminated $SrTiO_3$ (CHO/T-STO) and $CaHfO_3$/SrO-terminated $SrTiO_3$ (CHO/S-STO), respectively. **c, d,** B-site atom displacement vector maps of CHO/T-STO and CHO/S-STO, respectively. These maps show that atom displacements near both interfaces are tiny (0.06 and 0.11 Å for CHO/T-STO and CHO/S-STO, respectively, according to a large-range statistics), and their directions are irregular.

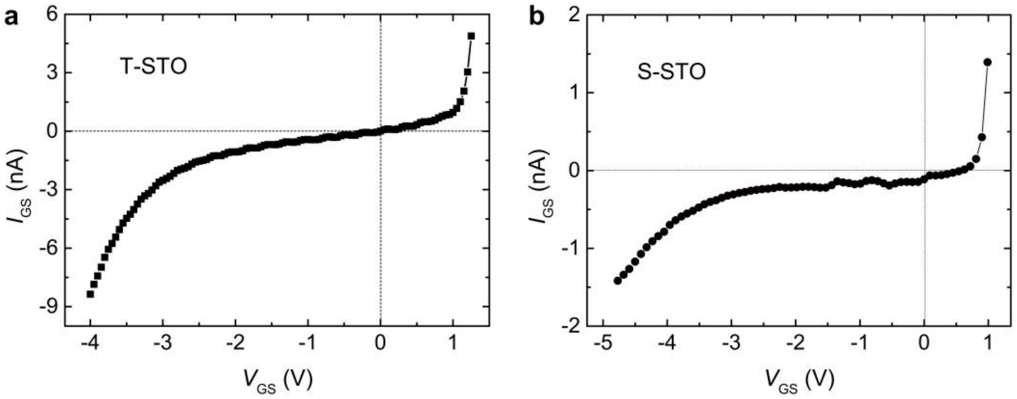

Supplementary Figure 12 | Gate current (I_{GS}) versus gate voltage (V_{GS}). Taken on the same devices as demonstrated on Fig. 3 in the main text. **a,** $CaHfO_3/TiO_2$-terminated $SrTiO_3$ (CHO/T-STO). **b,** $CaHfO_3$/SrO-terminated $SrTiO_3$ (CHO/S-STO). Throughout the study the I_{GS} was much smaller than the drain-source current I_{DS}.

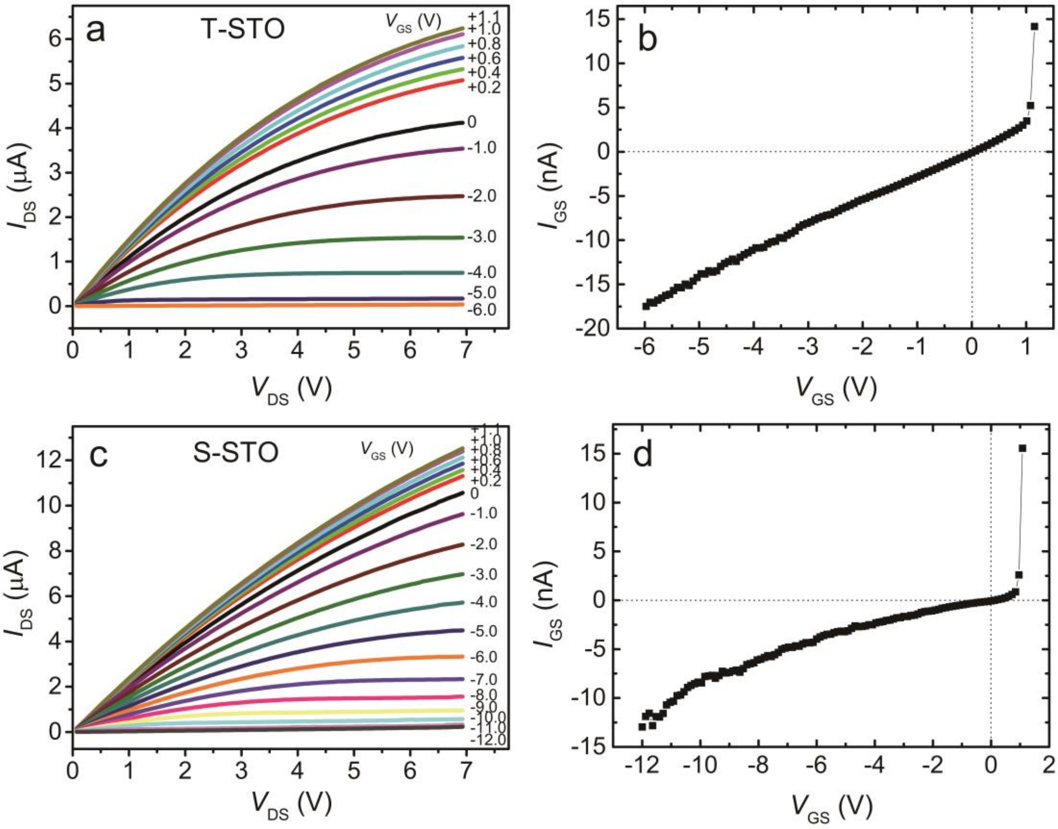

Supplementary Figure 13 | Field-effect transistors with in situ annealed 15 uc $CaHfO_3/SrTiO_3$ (CHO/STO) heterointerfaces. a and **b**, for TiO_2-terminated STO (T-STO). **c** and **d**, for SrO-terminated STO (S-STO). The data were measured at room temperature.

Supplementary References.

1. Brinkman, A. *et al*. Magnetic effects at the interface between non-magnetic oxides. *Nat. Mater.* **6**, 493–496 (2007).
2. Fuchs, D. *et al*. Incipient localization of charge carriers in the two-dimensional electron system in $LaAlO_3/SrTiO_3$ under hydrostatic pressure. *Phys. Rev. B* **92**, 155313 (2015).
3. Lin, W. N. *et al*. Electrostatic Modulation of $LaAlO_3/SrTiO_3$ Interface Transport in an Electric Double-Layer Transistor. *Adv. Mater. Interfaces* **1**, 1300001 (2014).
4. Lee, M., Williams, J. R., Zhang, S., Frisbie, C. D. & Goldhaber-Gordon, D. Electrolyte gate-controlled kondo effect in $SrTiO_3$. *Phys. Rev. Lett.* **107**, 256601 (2011).
